# Current Breast Cancer Screening Scenario in Brazil

**DOI:** 10.1055/s-0039-3399550

**Published:** 2019-11

**Authors:** Daniel Guimarães Tiezzi, Fleury Leonardo Orlandini, Hélio Humberto Angotti Carrara, Francisco José Cândido dos Reis, Jurandyr Moreira Andrade

**Affiliations:** 1 Division of Gynecologic Oncology and Breast Disease, Department of Gynecology and Obstetrics, Universidade de São Paulo, Ribeirão Preto Medical School, Ribeirão Preto, SP, Brazil

Breast cancer incidence has been substantially increasing in developing countries in the last decades.^1^ In Brazil, the total number of new diagnosed cases reaches 60,000 a year, resulting in an incidence rate of 60/100,000 women per year.^2^ Despite the high incidence, breast cancer is described as a relatively good prognosis cancer. The worldwide incidence/mortality rate is 3.3.^3^ However, this ratio varies within different populations with an evident decrease in developing countries when comparing to developed ones. The impact in prognosis in this scenario may be attributed to late diagnosis and discrepant technological improvement on cancer therapies.^4^

Mammographic screening has been described as an effective method for early breast cancer detection with substantial impact on breast cancer specific survival. A Norwegian study showed the implementation of a breast cancer screening program was able to detect an increased number of ductal carcinomas in situ (DCISs), and substantially reduced the number of locally advanced and metastatic tumors, resulting in a significant improvement in the breast cancer specific prognosis.^5^ They observed the fact of inviting women to mammography screening was the most significant factor impacting survival. This result has demonstrated that breast cancer awareness is a crucial piece of a breast cancer screening program.^6^

**Fig. 1:**
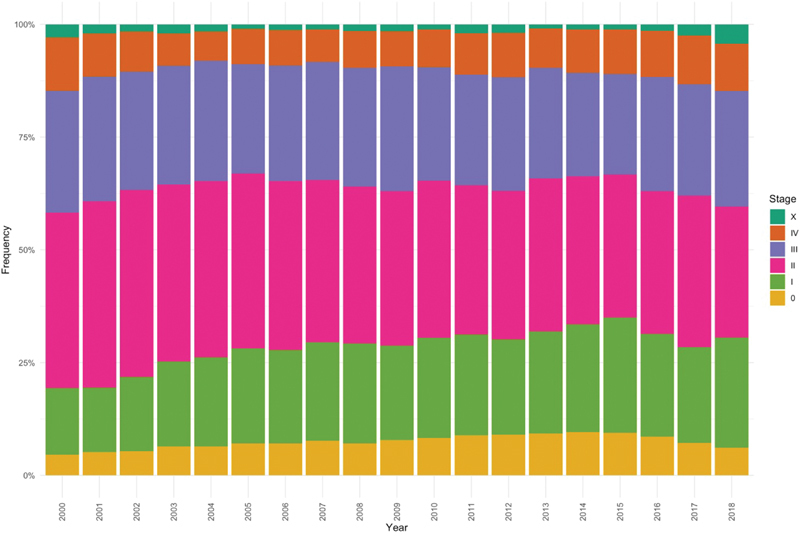
Breast cancer distribution according to stage in the state of São Paulo, Brazil. Source: FOSP - Fundação Oncocentro de São Paulo.^8^

The National Institute of Cancer in Brazil has issued an official recommendation for breast cancer screening in 2004, which was updated in 2015. It recommends mammography biennially for women aged 50 to 69 years old among the general population.^7^ However, it did not establish a standardized screening program, configuring an opportunistic one. Analyzing publicly available data from the Fundação Oncocentro de São Paulo (FOSP, in the Portuguese acronym), the organization responsible for compiling cancer data registry from the public health system across the State of São Paulo,^8^ it is possible to figure out how catastrophic is the result of the current breast cancer screening program in Brazil. Fig. 1 demonstrates the distribution of breast cancer stages from 2000 to 2018 in the group of women eligible for mammography screening (from 50 to 69 years old). Note that ∼ 40% of the women are diagnosed with locally advanced or metastatic breast cancer. This scenario is strikingly distinct from the data in the Norwegian population before the implementation of the breast cancer screening program (Table 1).

**Table 1:** The prevalence of invasive breast cancer according to stage in the state of São Paulo, Brazil from 2000 to 2017 and in the Norwegian population before the implementation of the national breast cancer screening program

Stage	Brazil (*n* = 22,527)	Norwegian (*n* = 26,883)
I	21.3%	48.5%
II	35.2%	38.5%
III	25.2%	5.3%
IV	8.9%	6.5%
X	1.6%	−

Combining data from the Brazilian Geography and Statistics Institute (IBGE, in the Portuguese acronym) and FOSP, we observed that from 2003 to 2013, the biennial mammography coverage increased by 10.5%. From 2000 to 2016, the proportions of in situ and localized tumors increased 6.9% and 3.9%, respectively. During the same period, the proportions of regional and distant tumors decreased by 7.1% and 4%, respectively.^9^ However, this scenario is far away from an acceptable level in a R$ 2.2 trillion GDP federated state.^10^

**Fig. 2:**
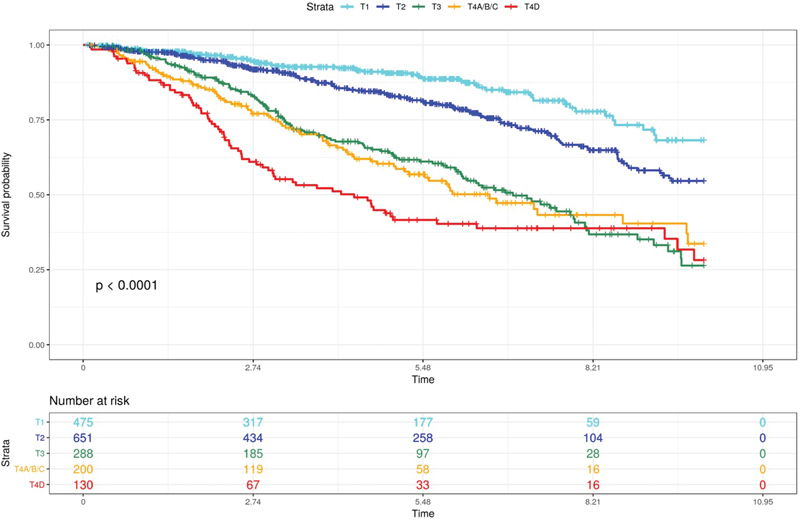
10-year breast cancer specific survival among 1,752 invasive non-metastatic breast carcinoma patients according to T stage. *Data from an unpublished study approved by the local Committee in Ethics number 2.638.453/2018.

The impact of late diagnosis in breast cancer is remarkable. Analyzing data from a public reference hospital in Ribeirão Preto, state of São Paulo, Brazil, including all breast cancer patients from 2000 to 2013 (*n* = 1,955), we observed a similar scenario. A total of 1,025 patients were diagnosed with locally advanced or metastatic disease, and 34.5% of them presented with a tumor > 5 cm (T3) in diameter or as T4 stage (skin/chest wall involvement or inflammatory breast cancer). Fig. 2 shows the 10-year disease specific survival according to the T stage among this population. Note how worse is the disease specific survival among T3 and T4 stage patients (non-published data). Cancer is a progressive disease, thus late stage diagnosis is usually due to the lack of patient awareness or the delay on patient referral and diagnosis/treatment. It's not clear how each of these factors contributes to the current scenario. What is clear is that effective measures should be implemented as soon as possible.^11^ The implementation of a structured breast cancer screening program, including a routine invitation to eligible women to participate and the offer of a fast track access for patients with suspicious clinical or subclinical breast lesions, is crucial for early stage breast cancer diagnosis.
